# GLUT1 and TUBB4 in Glioblastoma Could be Efficacious Targets

**DOI:** 10.3390/cancers11091308

**Published:** 2019-09-05

**Authors:** Maheedhara R. Guda, Collin M. Labak, Sara Ibrahim Omar, Swapna Asuthkar, Subra Airala, Jack Tuszynski, Andrew J. Tsung, Kiran K. Velpula

**Affiliations:** 1 Department of Cancer Biology and Pharmacology, University of Illinois College of Medicine at Peoria, Peoria, IL 61605, USA; 2 Department of Oncology, University of Alberta, Edmonton, AB T6G 1Z2, Canada; 3 Department of Health Professions, Rollins College, Winter Park, FL 32789, USA; 4 Department of Neurosurgery, University of Illinois College of Medicine at Peoria, Peoria, IL 61605, USA; 5 Illinois Neurological Institute, Peoria, IL 61605, USA; 6 Department of Pediatrics, University of Illinois College of Medicine at Peoria, Peoria, IL 61605, USA

**Keywords:** TUBB4, GLUT1, CR-42-24, homology modeling, fasentin

## Abstract

Glioblastoma multiforme (GBM) is the most aggressive and deadly brain tumor, portending a median 13-month survival even following gross total resection with adjuvant chemotherapy and radiotherapy. This prognosis necessitates improved therapies for the disease. A target of interest for novel chemotherapies is the Warburg Effect, which describes the tumor’s shift away from oxidative phosphorylation towards glycolysis. Here, we elucidate GLUT1 (Glucose transporter 1) and one of its associated binding partners, TUBB4 (Tubulin 4), as potentially druggable targets in GBM. Using data mining approach, we demonstrate that GLUT1 is overexpressed as a function of tumor grade in astrocytoma’s and that its overexpression is associated with poorer prognosis. Using both mass spectrometry performed on hGBM (human glioblastoma patient specimen) and in silico modeling, we show that GLUT1 interacts with TUBB4, and more accurately demonstrates GLUT1’s binding with fasentin. Proximity ligation assay (PLA) and immunoprecipitation studies confirm GLUT1 interaction with TUBB4. Treatment of GSC33 and GSC28 cells with TUBB4 inhibitor, CR-42-24, reduces the expression of GLUT1 however, TUBB4 expression is unaltered upon fasentin treatment. Using human pluripotent stem cell antibody array, we demonstrate reduced levels of Oct3/4, Nanog, Sox2, Sox17, Snail and VEGFR2 (Vascular endothelial growth factor receptor 2) upon CR-42-24 treatment. Overall, our data confirm that silencing TUBB4 or GLUT1 reduce GSC tumorsphere formation, self-renewal and proliferation in vitro. These findings suggest GLUT1 and its binding partner TUBB4 as druggable targets that warrant further investigation in GBM.

## 1. Introduction

Glioblastoma (GBM) is a WHO (World Health Organization) Grade IV diffuse glioma with a 9.8% 5-year survival rate. Given the standard of care, that include gross total resection with adjuvant radiation and chemotherapy, the prognosis still necessitates for better treatment options for patients with the devastating astrocytic tumor [1]. Treatment of GBM is difficult because of its intrinsic heterogeneity, both at the cellular and the molecular levels. Recent investigations revealed the existence of glioblastoma stem cells (GSCs), responsible for tumor initiation and maintenance, ensnare GSCs as significant targets to develop novel therapeutics for glioblastoma [2]. This underscores a critical need to identify avenues and distinguish new targets for creating compelling strategies against GBM.

Tumor metabolism has not been studied as a significant driver of glioblastoma tumorigenesis until recently. To support the rapid cell progression and proliferation, these tumors utilize glycolysis as opposed to mitochondrial oxidative phosphorylation (OXPHOS). The tumor cells even with aerobic conditions switch to glycolysis, leading to a phenomenon termed as the Warburg effect. This Warburg effect, a shift from oxidative phosphorylation to glycolysis for primary energy derivation, has become a recent target of interest in normalizing GBM’s metabolic phenotype [3,4]. Contemporary efforts in reversing the Warburg effect specific to GBM have been focused on inhibiting various glycolytic enzymes, including hexokinase [5], pyruvate kinase [6] and pyruvate dehydrogenase kinase [7]. Targeting these glycolytic enzymes in GBM has been shown to attenuate tumor growth, increase apoptosis and reduce tumor invasion in vitro [8], but these targets still fall short in in vitro and preclinical trials. Since metabolic adaptations are a characteristic of GBM, it is possible that a therapeutic strategy of targeting abnormal metabolism and identification of new targets to reverse the Warburg effect could be effective in treating GBM.

The first step of glycolysis prior to any modification of the glucose molecule is its influx from the extracellular space into the cellular cytosol. This process is mediated by a family of unidirectional transport proteins that are collectively known as the glucose transporter (GLUT) family [9]. GLUT1 is one such primary mediator of glycolysis and its availability is a rate-limiting step in the glycolytic process. Interestingly, GLUT1 has been found to be significantly up regulated both in vitro and in vivo GBM paradigms [10,11]. This suggests that the blockade of GLUT1 may prove efficacious in reversing the Warburg effect in GBM, particularly if it can be targeted in a tissue-specific fashion that avoids the blockade of GLUT1 throughout non-neoplastic tissue. Fasentin, a recently developed small molecule, is a GLUT1-specific competitive inhibitor that has been shown to induce cell death in limited applications [12]. Thus, a specific inhibitor for our target of interest, GLUT1, needs to be tested to assess its efficacy in reversing the Warburg effect in in vitro GBM models.

Recent evidence showed that the upregulation of GLUT1 is facilitated in part by tubulin-dependent membrane trafficking [13]. In the previous few years, novel colchicine derivatives, which inhibit α/β-tubulin nucleation, are synthesized that demonstrated anti-cancer activity [14]. The primary cytotoxicity that such molecules confer is generally agreed upon as an inhibitor of mitosis due to impaired spindle formation—colchicine and its derivatives are known as “spindle poisons” [15]. There are two loci on microtubules where these derivatives bind, both of which are found on the β-tubulin monomer [16].

Our preliminary data demonstrate a strong association between GLUT1 and tubulin4 (TUBB4) as observed in immunoprecipitated, mass spectrometric analysis and it is plausible that GLUT1 can be downregulated via tubulin-mediated trafficking inhibition. Due to recent advents in the development of novel colchicine derivatives, we considered the possibility of downregulating GLUT1 via colchicine-induced microtubule destabilization to ultimately reduce the Warburg effect in GBM in hopes of attenuating the phenotypic hallmarks of cancer.

Here, we set out to demonstrate GLUT1 as a bonafide target in GBM, and that it can be targeted via its association with TUBB4. We show that both GLUT1 and TUBB4 are expressed in human-derived GBM samples and their high expression correlates with poorer prognosis as evidenced by data mining approach and immunohistochemical staining. The homology binding studies predicted the binding of GLUT1 and TUBB4, while the proximity ligation assay and immunoprecipitation studies confirmed their association. Fasentin, an inhibitor of GLUT1 and CR-42-24, colchicine derivative and inhibitor of tubulin were studied to demonstrate their association in the mesenchymal subtyped glioblastoma stem cells GSC33 and GSC28. Silencing TUBB4 using CR-42-24 and GLUT1 by using fasentin, decreased GSC cell viability, tumorsphere formation and stemness in vitro.

## 2. Methods

### 2.1. Cell Culture and Reagents

In this study, we used two different GBM GSC cell lines GSC33 and GSC28. Both these cell lines are established mesenchymal subtypes and authenticated as previously reported [17,18]. They were obtained from the MD Anderson Cancer Center (Dr. Bhat’s Laboratory) and University of Wisconsin (Dr. John Kuo’s Laboratory). These GSC were grown in DMEM/F12 supplemented with B27 (Life Technologies, Carlsbad, CA, USA), Epidermal growth factor (EGF) (20 ng/mL; Millipore, Sigma, St. Louis, MO, USA), basic fibroblast growth factor (bFGF) (20 ng/mL; Millipore, Sigma) and 1% penicillin/streptomycin (Life Technologies). CR-42-24 is a novel colchicine-derived inhibitor developed by Dr. Jack Tsuzynski’s lab (University of Alberta, Edmonton, AB, Canada) and fasentin was obtained from Millipore Sigma. All the primers used in the study were obtained from IDT (Coralville, Iowa, IA, USA). GLUT1 antibodies were obtained from Novus (Centennial, CO, USA) and Abcam (Eugene, OR, USA); TUBB4 antibodies were obtained from Santa Cruz and Abcam and GAPDH (Glyceraldehyde 3-phosphate dehydrogenase) antibody was obtained from Santa Cruz (Santa Cruz, CA, USA). A Proximity Ligation Assay kit was obtained from Sigma (Duolink^®^ In Situ Red Starter Kit Mouse/Rabbit; DUO92101). Protein G beads were obtained from Miltenyi Biotech (San Diego, CA, USA). The MTT reagent and human pluripotent stem cell antibody array (ARY010) was from R & D Systems (Minneapolis, MN, USA).

### 2.2. TCGA Cohort Analysis

The mRNA expression and the clinical data from 172 cases were obtained from the glioblastoma cohort using the TCGA (The Cancer Genome Atlas) data portal (http://cancergenome.nih.gov/). We also used project Betastasis (http://www.betastasis.com) to obtain the mRNA expression and survival data from the Repository for Molecular Brain Neoplasia Data (REMBRANDT), a publically available dataset. Glioblastoma Bio Discovery Portal was used to calculate the expression of GLUT1 between the subtypes.

### 2.3. Immunohistochemical Analyses of hGBM (Human Glioblastoma Patient) Specimens

The human glioblastoma (hGBM) surgical biopsy specimens were obtained from Saint Francis Medical Center (Peoria, IL, USA) and processed in accordance with the University of Illinois College of Medicine at Peoria (UICOMP) Institutional Review Board–approved protocols (Protocol #85193). Representative sections of GS4, GS6-4, GS11, GS18 and GS22 specimens all confirmed to be GBM by an independent neuropathologist at our institution were DAB (3,3′-Diaminobenzidine) stained with anti-GLUT1 and anti-TUBB4 antibodies as described previously [19]. For DAB immunohistochemistry, sections were probed with the aforementioned antibodies and then stained with DAB and further stained with hematoxylin. Negative controls were maintained without primary antibody by using an IgG antibody. The sections were then visualized using a confocal microscope according to standard protocols.

### 2.4. Immunoblot, Immunoprecipitation, Stem Cell Array and RT-PCR Analysis

Total protein extracts from various treatments were resolved by 10% SDS-PAGE (sodium dodecyl sulfate- polyacrylamide gel electrophoresis) transferred to nitrocellulose membrane and were blocked in Tris-HCl, pH 7.5, 500 mM NaCl, 0.1% Tween 20 and 5% nonfat milk. Then blots were incubated with GLUT1 (1:500; Novus Bio, CO, USA), TUBB4 (1:1000; Abcam) and GAPDH (1:1000; Santa Cruz Biotechnologies) primary antibodies, and subsequently incubated with 1:2000 dilution of species-specific, horseradish peroxidase (HRP)-conjugated secondary antibody. Imaging and data analysis were carried out using enhanced chemiluminescence (ECL) Western blotting detection reagents on Hyperfilm-MP autoradiography film. The GAPDH antibody was used to verify equal loading of proteins in all lanes. Approximately 300 µg of total protein cell lysates from both untreated and 1µM CR-42-24 GSC28 cells were incubated with 50 µL of protein G beads (Miltenyi Biotech) followed by 2 µg of TUBB4 antibody with end-to-end rotation overnight as described previously [20]. The obtained eluates were verified for the expression of GLUT1 and TUBB4. IgG is shown as a loading control. Around 300 µg of control and 1 µM CR-42-24 treated total cell lysates from GSC28 cells were subjected to human pluripotent stem cell array by following the manufacturer’s instructions. RT-PCR was conducted from the total RNA isolated from the hGBM specimens using the SYBR green method. The primers used in the study were GLUT1 sense 5′-TCGTCGGCATCCTCATCGCC-3′ and antisense 5′-CCGGTTCTCCTCGTTGCGGT; GAPDH sense 5′-AATCCCATCACCATCTTCCA-3′ and antisense 5′-TGGACTCCACGACGTACTCA-3′. The original pictures of Western blotting can be found in Supplementary Materials.

### 2.5. Mass Spectrometry

Approximately, 400 µg of total protein obtained from GSC33 cell lysates were incubated with an anti-GLUT1 primary antibody (2 µg) overnight at 4 °C on an end-to-end rotator. Bound GLUT1 fractions were collected from µMACS protein G microbeads and the extracts were used for LC–MS/MS spectra analysis of immunoprecipitated extract proteins. The sample was cleaned with G-Biosciences Perfect Focus, followed by thermo pierce detergent removal chromatography, then digested with trypsin and analyzed by Liquid Chromatography/Mass Spectrometry. The results were searched against the human database and the obtained spectra were analyzed by using the Mascot Server (University of Illinois, Champaign-Urbana, Illinois, IL, USA). The cut off for these putative identified proteins were set to 99% identity.

### 2.6. Proximity Ligation Assay

For this experiment, we used the Duolink^®^ In Situ Red Starter Kit (Mouse/Rabbit; DUO92101; Sigma) according to the manufacturer’s recommendations. Both untreated and 1 µM CR-42-24 treated GSC33 cells were plated onto 8-well laminin-coated chambered slide. The cells were washed with PBS and fixed in 3.7% paraformaldehyde for 30 min on ice. After washing with PBS, the cells were permeabilized with 0.3% Triton X-100 in PBS for 5 min at room temperature. To reduce the nonspecific signal, the cells were incubated with a blocking solution for 1 h at 37 °C. Then, primary 1× antibody diluent with two primary antibodies (GLUT1 and TUBB4) were added to the cells and incubated overnight at 4 °C. The negative control was performed by only one primary antibody (rabbit polyclonal antibody) into cells for incubation at 4 °C overnight. The following day, after washing, the cells were incubated with the diluted proximity ligation assay (PLA) probes as per the manufacturer’s instructions. Cells were washed twice for 5 min with wash buffer A (from kit), followed by incubation with the ligation reaction mix for 30 min at 37 °C. Cells were washed twice for 2 min with wash buffer A, and amplification was performed by incubating cells with polymerase reaction mix for 100 min at 37 °C. Cells were washed twice for 10 min with wash buffer B (from kit) and once for 10 min with 0.01× wash buffer B. Coverslips were mounted onto slides with ProLong Gold mounting medium containing DAPI. The images of the cells were acquired using an Olympus BX61 microscope (Olympus BX61 Fluoview, Minneapolis, MN, USA).

### 2.7. TUBB4-GLUT1 Complex Prediction

We used two models of the GLUT1 protein. The first was the X-ray crystallographic structure of GLUT1, which is missing the cytoplasmic residues 1 to 8 and 456 to 492 (protein data base (PDB) ID 4PYP) [21]. The second structure was a homology model of the entire GLUT1 protein. The template for the homology model was the x-ray resolved crystallographic structure, PDB ID 5EQH [22]. The missing residues from the template were modeled using the loop modeler application in the molecular operating environment (MOE) software. The TUBB4 model was created in MOE using UniProt accession number P68371 as the target sequence [23] and PDB ID 4U3J as the template structure. We used MOE to perform protein–protein docking of TUBB4 to the cytoplasmic domain of GLUT1, which is constituted of residues 1–11, 88–90, 145–155, 207–271, 329–334, 389–401 and 451–492 [23]. We calculated the partial charges of the two proteins using MOE. It was observed that TUBB4 had a negatively charged C-terminus. Four docking simulations were performed each of which was set to output the top 10 ranked models. While the protein was rigid in the initial docking calculations, the residue side-chains were allowed to be flexible at the model refinement stage. For the first two docking simulations, each GLUT1 model was set as the receptor with the whole cytoplasmic domain of the protein chosen as a possible binding site, while TUBB4 was set as the ligand. The possible binding residues for the second set of calculations comprised residues 10–11, 88–92, 153–154, 205–218, 221–232, 241, 244–251, 253–257, 330, 397, 398 and 400 of TUBB4, which were at the negatively charged domain of the protein. These were docked to 4PYP structure and the generated homology model.

Twelve of the forty predicted complex models were excluded since TUBB4 was partly interacting with a membrane-bound region of GLUT1 in those models. Another two models from docking were also excluded since TUBB4 was predicted at that position only once. Interestingly, TUBB4 was predicted to be in similar positions in nine of the forty complexes. The least binding energy conformation of the GLUT1-TUBB4 complex was chosen for further analysis. This complex obtained from protein–protein docking was protonated in MOE then parameterized using the Amberff14SB force field and solvated in a 12 Å water box. Since GLUT1 is a membrane bound protein, we simulated the protein with restraints on residues 12–33, 67–87, 91–112, 121–144, 156–176, 186–206, 272–293, 307–328, 335–355, 366–388, 402–422 and 430–450 that constitute the transmembrane domain of the protein. The molecular dynamics (MD) simulation was run for a short time period of 50 ns.

### 2.8. GLUT-Fasentin Mode of Binding Prediction

In order to predict the mode of binding of fasentin (PubChem CID 879520) to GLUT1, four different docking programs (Autodock, Vina, DOCK6 and GLIDE SP) were used following a consensus approach. The four programs are based on different algorithms regarding their placement methods as well as their scoring functions: Autodock [24] includes a Lamarckian genetic algorithm with an empirical scoring function, which has proved successful in several case studies [25,26]. Vina [27] provides a combination of empirical and knowledge-based scoring functions with an iterated local search global optimizer that turned out to improve the accuracy of the binding mode prediction compared to Autodock. DOCK6 [28] makes use of an anchor-and-grow algorithm and a grid-based score based on non-bonded terms of a molecular mechanic force field. Finally, Glide SP [29], available within the Schrödinger software, applies a series of hierarchical of filters to search for possible locations of the ligand and uses the OPLS-AA (Optimized Potentials for Liquid Simulations) force field to score the final poses. Docking was performed from the crystal structure of the human GLUT1 (PDB ID 4PYP) [21]. Prior to that, the Protein Preparation Wizard of the Schrödinger software [30] was applied to assign position-optimized hydrogen atoms to the protein structure, predict protonation states at neutral pH using the PROPKa algorithm and eventually minimize the energy of the entire protein. The ligand structure was prepared using the LigPrep tool [30] of the Schrödinger package, which enables the generation 3D structures for each ionization state, tautomer and stereoisomer of the compound. As far as fasentin is concerned, no structural isomers were found and only one protonation state was identified at neutral pH. Each docking software used requires the definition of a box with limited size (less than 50 Å typically), which includes the binding pockets to be investigated. In order to identify those pockets, the Site Finder tool available within the MOE software was used. It was determined that the most probable binding site (propensity for ligand binding (PLB) score = 3.69) is located within the channel and includes 69 residues, as depicted in Figure 4A. A less probable binding site (PLB = 0.39) including 13 residues was found on the surface of the GLUT1 structure next to the entrance of the channel. The grid box used for the docking step was defined so it included all the residues of both identified binding sites (the residue closest to the center of the box corresponds to methionine (MET) 142, whereas the dimensions of the box have been set to 40 Å in each direction x, y and z, see Figure 4A). For each docking program, i.e., Autodock, Vina, DOCK6 and GLIDE SP, a maximum of 20 possible poses of fasentin were generated. A short 2 ps in vacuo minimization of each pose was then performed by restraining the protein atoms. Eventually, all the poses were clustered using the cpptraj tool of the Amber package [31]. A hierarchical-agglomerative approach was applied following the average-linkage rule to estimate the intercluster distance. As the protein structure is kept fixed during the docking process, clustering analysis was done based on the RMSD (Root Mean Square Deviation) of the ligand atoms setting a RMSD threshold of 2.0 Å.

### 2.9. MTT Assay

Both GSC33 and GSC28 cells were treated with increased concentrations of 5 µM, 25 µM, 50 µM fasentin and 0.25 µM, 0.5 µM, 1 µM CR-42-24 for 24 h. Cell viability was measured using the MTT (3-[4,5-dimethylthiazol-2-yl]-2,5 diphenyl tetrazolium bromide) assay according to the manufacturer’s instructions (R & D Systems).

### 2.10. Limiting Dilution Assay and Tumorsphere Formation Assay

For this assay, a 96-well plate pre-coated with poly D-lysine was used to plate GSC33 in increasing cell numbers (500, 1000 and 2000 cells/well) with 12 replicates/cell number. Prior to plating, the spheres of cells were disassociated into single-cell suspension. Cells were treated with 500 nM, 1 µM CR-42-24 and 25 µM and 50 µM fasentin. Both untreated and treated GSC were counted on 1st, 7th and 14th day under a phase contrast microscope, and data were analyzed using the extreme limited dilution analysis (ELDA) platform to determine stem cell frequency (http://bioinf.wehi.edu.au/software/elda/) [32]. The tumorsphere formation assay was performed as described by Li et al. [33]. For this experiment, GSC33 was dissociated into single cells and plated on a poly D-lysine coated 96 well plate. The cells were allowed to grow for 14 days and all the tumorspheres from each well (both control and varying concentrations of fasentin and CR-42-24) were counted under a microscope (*n* = 6). 

### 2.11. Immunofluorescence Analysis

GSC28 were seeded on 8-well chamber slides containing both control and 1 µM CR-42-24 for 24 h. GSC28 cells were fixed with 10% buffered formalin phosphate and incubated with 1% bovine serum albumin in PBS at room temperature for 1 h to avoid non-specific staining. After the slides were washed with PBS, anti-GLUT1 and anti-TUBB4 antibodies were added at a concentration of 1:500. The slides were incubated overnight at 4 °C and washed three times with PBS to remove excess primary antibody. Cells were then incubated with Alexa Fluor^®^ 594 (red) for GLUT1 and Alexa Fluor^®^ 488 (green) fluorescent-labeled secondary antibodies for 1 h at room temperature. The slides were then washed another three times with PBS (Phosphate-buffered saline), treated with DAPI((4′,6-diamidino-2-phenylindole), covered with glass coverslips and fluorescent photomicrographs were obtained.

### 2.12. Statistical Analysis

The results shown are represented as mean ± SD. Graph pad 8.0 was used to perform student’s *t*-tests and ANOVA to evaluate the differences between the control and treated groups. For multiple comparisons within TCGA and REMBRANDT, we used Student’s *t*-test to calculate the expressional differences between glioblastoma and normal brain samples. All *p*-values were considered statistically significant with a value <0.01. Logrank *p* value was calculated in Project Betastasis [34].

## 3. Results

### 3.1. GLUT1 is Overexpressed in hGBM and Negatively Correlates with Patient Prognosis

**Figure 1:**
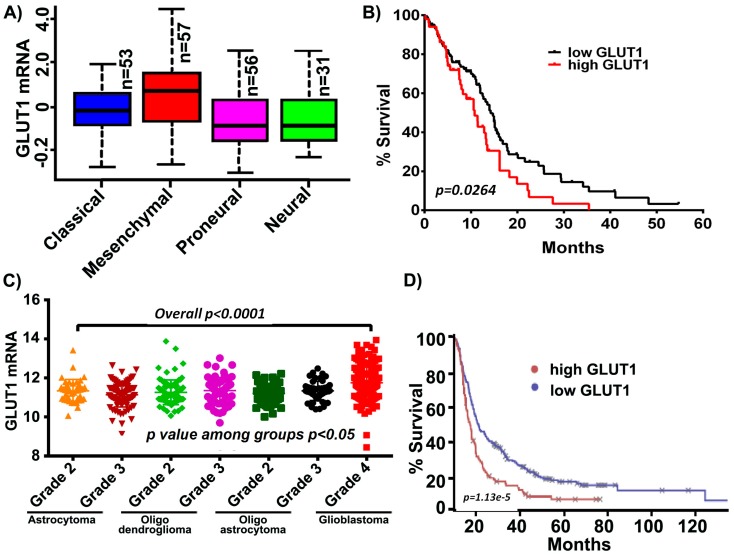
GLUT1 expression in The Cancer Genome Analysis (TCGA) and Repository for Molecular Brain Neoplasia Data (REMBRANDT) cohort. (**A**) GLUT1 expression is high in the mesenchymal subtype compared to other glioblastoma multiforme (GBM) subtypes. (**B**) Kaplan-Meier curves show increased GLUT1 corresponds to decreased survival. (**C**) GLUT1 is highly expressed in GBM relative to lower grade glioma (*p* < 0.0001). (**D**) Survival analysis of long-term survivors from the REMBRANDT dataset show that the patients with high GLUT1 expression show shorter overall survival.

Increased levels of GLUT1 have been demonstrated in GBM and its inhibition has been shown to increase the effectiveness of temozolomide, a current standard of care in GBM [35]. It is also reported that tumor cells from the enhancing region showed high expression of mesenchymal genes including GLUT1 in the necrotic/hypoxic region [36]. We, therefore, started by assessing if an increase in GLUT1 expression was characteristic in GBM patients by using a data mining approach. Using the data obtained from the TCGA GBM cohort and glioblastoma bio discovery portal [37], we compared the mRNA expression of GLUT1 across GBM subtypes. Of these subtypes, the mesenchymal subtype demonstrated the highest mRNA levels of GLUT1, in comparison to proneural, classical and neural subtypes (Figure 1A). Next, when the aggregated patient population from the GBM dataset was dichotomized into those who expressed more GLUT1 than the sample median and those who expressed less GLUT1 than the median, the high-GLUT1 group demonstrated worse survivorship (Figure 1B). Next, we assessed the expression of GLUT1 across all the WHO grade diffuse glioma (astrocytoma, oligodendroglioma, oligoastrocytoma and glioblastoma). High expression levels of GLUT1 (*p* < 0.0001) were observed in glioblastoma when compared to the low grade tumors, indicative of GLUT’s possible role in tumor aggressiveness (Figure 1C). Using the Project Betastasis, we next examined the clinical outcomes of GLUT1 in long-term GBM survivors. The Kaplan–Meier data obtained from the REMBRANT glioblastoma cohort of 329 cases showed that patients with high GLUT1 expression had a shorter overall survival when compared to the low GLUT1 expression. This data suggest that GLUT1 may be potential target in glioblastoma, however it is not clear if the expression of GLUT1 contributes to the mesenchymal subtype (Figure 1D).

### 3.2. hGBM Specimens Demonstrate Increased GLUT1 Expression

**Figure 2:**
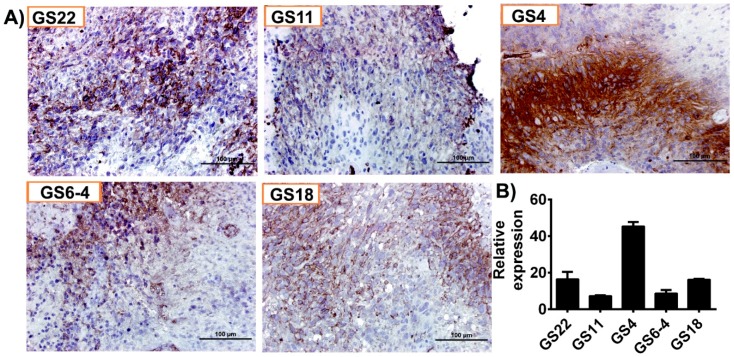
GLUT1 is highly expressed in human glioblastoma (hGBM) surgical biopsies. (**A**) Immunohistochemical staining for GLUT1 with anti-GLUT1 antibody (brown, diaminobenzidine; light blue, nuclear counter stain with DAPI; GS = glioblastoma specimen). (**B**) RT-PCR analysis of GLUT1 from hGBM specimens. GAPDH is used as a loading control.

Immunohistochemical analysis conducted on five hGBM specimens from our own cohort of samples obtained as per the approved IRB protocol, showed increased positivity for GLUT1 (Figure 2A). Of these specimens, GS4 and GS22 showed increased positivity, whereas GS6-4, GS18 and GS11 were stained moderately. Next, we isolated total RNA from the aforementioned hGBM samples and qRT-PCR analysis confirmed GLUT1 expression in all the specimens tested in this study (Figure 2B).

### 3.3. GLUT1 Derived from hGBM Demonstrate its Association with TUBB4

**Figure 3:**
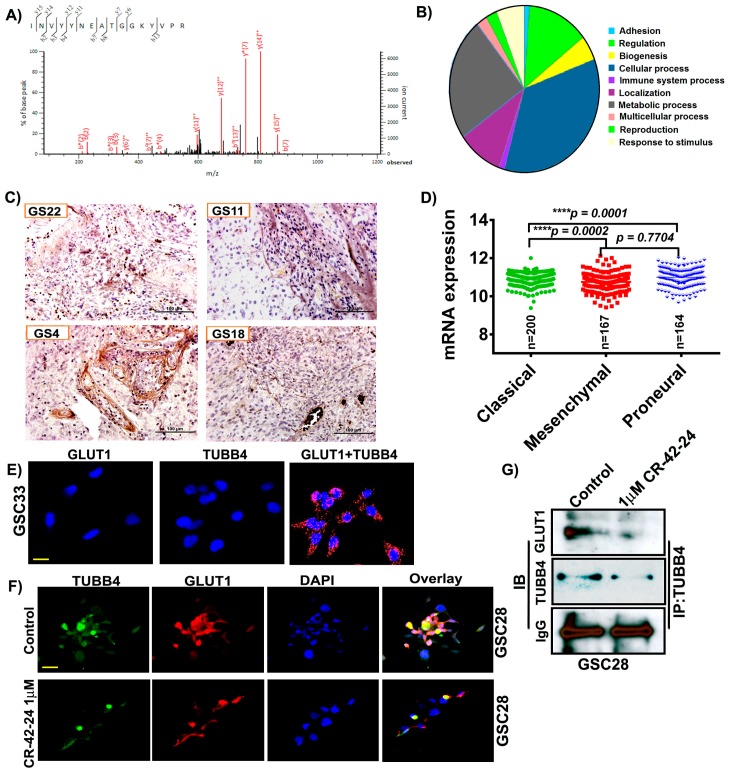
GLUT1 associate and interact with TUBB4 in GSC. (**A**) Around 400 μg of total protein was extracted from hGBM specimen (GS6-4). The lysates were immunoprecipitated with GLUT1 antibody and the eluates were subjected to LC/MS analysis. LS/MS spectra showed the high relative intensity of reporter ions for TUBB4. The spectra acquisition is mentioned in the methods section. (**B**) Annotation of the spectral data using Pantherdb software. (**C**) Immunohistochemical analysis for TUBB4 using an anti-TUBB4 antibody (brown, diaminobenzidine; light blue, nuclear counter stain with DAPI; GS = glioblastoma specimen). (**D**) TCGA analysis of TUBB4. (**E**) The proximity ligation assay (PLA) confirmed the association of GLUT1 and TUBB4 in GSC33 cells. The representative micrograph of GLUT1 + TUBB4 shows the positive PLA. Red spots confirmed the association. Single antibody stain with TUBB4 and GLUT1 showed negative results. Scale = 10 µm. (**F**) Immunofluorescence analysis show a TUBB4-GLUT1 association in GSC28 control cells. CR-42-24 treatment diminished their association. Blue = DAPI; red = GLUT1; green = TUBB4; yellow color in the overlay shows the association. Scale = 10 µm. (**G**) Immunoprecipitation of GSC28 control and 1 uM CR-42-24 treated cells confirmed the interaction of GLUT1-TUBB4. IgG is shown as a loading control.

To identify the binding partners and possible regulators of GLUT1, we performed LC/MS analysis on a GS4 hGBM specimen. Annotation of the LC/MS result was performed on the Mascot server and demonstrated a high probability of association with TUBB4 based on the ionic residues shown (Figure 3A). The binding score was calculated to be 23.2 with a binding energy of −0.0019. The TUBB4 sequence INVYYNEATGGKYVPR obtained upon GLUT1 binding is verified and was correlated to TUBB4 from NCBI. In silico analysis performed using pantherdb (www.pantherdb.org) software also verified GLUT1’s involvement in a variety of signaling pathways including cell adhesion (2%), metabolic process (28%) and cellular process (41%; Figure 3B). As our LC/MS confirmed the association of GLUT1-TUBB4, we next checked the expression of TUBB4 in our hGBM specimens using an immunohistochemical analysis. The staining results showed high expression of TUBB4 in GS22, GS11, GS4 and GS18 specimens (Figure 3C). TCGA analysis generated using mRNA expression of TUBB4 showed that TUBB4 expression is high in the mesenchymal subtype when compared to classical and proneural subtypes. These results are comparable to the expression profile of GLUT1 (Figure 3D). To further confirm the association of GLUT1 and TUBB4 we next asked if a proximity ligation assay (PLA) could detect a direct association between GLUT1 and TUBB4 in GSC33 cells. We observed a PLA signal of high intensity in the presence of both GLUT1 and TUBB4 (Figure 3E) confirming their association (red spots). The PLA for only GLUT1 or only TUBB4 showed no signal. We next performed the immunofluorescence experiment and observed that both TUBB4 and GLUT1 associated with each other in the untreated GSC28 cells and upon treatment with 1 µM CR-42-24, their expression levels were reduced and so was their association (Figure 3F). Next, we performed immunoprecipitation experiments to confirm the interaction between GLUT1 and TUBB4. GSC28 control and treated cells with 1 µM CR-42-24 were subjected to immunoprecipitation using the TUBB4 antibody. The immunoblot analysis performed on the eluates of TUBB4 immunoprecipitated samples using GLUT1 antibody confirmed their interaction. These results confirmed that GLUT1 and TUBB4 physically associated and interacted in GSC (Figure 3G).

### 3.4. Homology Binding and Docking Studies Confirm GLUT1-TUBB4 Association

Two GLUT1 protein structures were used as receptors in the protein–protein docking calculations. The first was the 4PYP [21] structure and the second was a homology model of the entire protein based on the 5EQH [22] structure. We ran a set of docking calculations in which the entire TUBB4 protein structure was set as a ligand and the cytoplasmic residues (1–11, 88–90, 145–155, 207–271, 329–334, 389–401 and 451–492) of each of the GLUT1 models were set as the receptor binding site. We also ran another set of docking calculations in which residues 10–11, 88–92, 153–154, 205–218, 221–232, 241, 244–251, 253–257, 330, 397, 398 and 400 of TUBB4, which were at the negatively charged domain of the protein were set as the ligand binding site. A total of forty models were obtained from the docking calculations; these were visually inspected. Twelve of the forty poses in which TUBB4 was predicted to partly interact with the transmembrane region of GLUT1 were excluded. Further analysis revealed one dominant pose, which was predicted nine times. The predicted pose with the highest affinity of TUBB4 to GLUT1 had a binding energy of – 60 kcal/moL. Although the side-chains of TUBB4 and GLUT1 were allowed to be flexible during the refinement stage of docking, their backbones were rigid, which could be a drawback. The highest affinity TUBB4-GLUT1 complex was, therefore, simulated in a water box with restraints on the transmembrane residues of GLUT1 for 50 ns. This short simulation would allow protein flexibility for better orientation of the two proteins.

**Figure 4:**
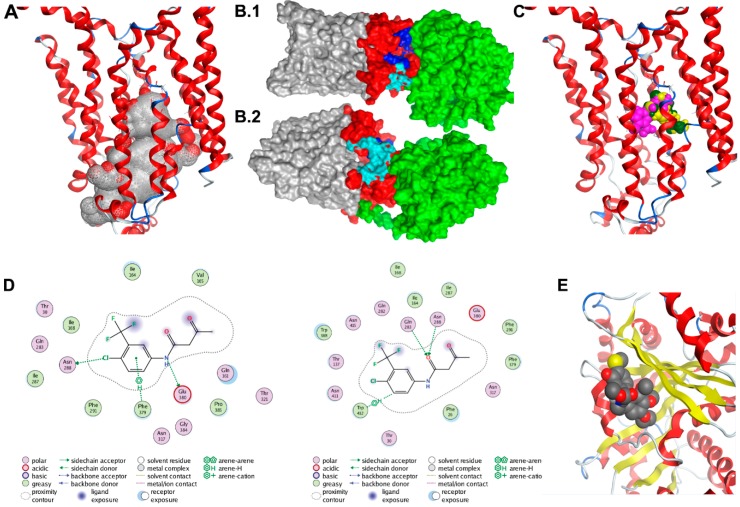
Docking studies of GLUT1-TUBB4. (**A**) Alpha spheres (grey and red spheres) obtained by applying molecular operating environment’s (MOE’s) Site Finder tool on the crystal structure of the human GLUT1 (Deng et al. 2014; PDB ID 4PYP) accounting for the most probable binding pocket. The binding region includes 69 residues, many of them being deeply buried within the channel. Contact atoms of the protein are highlighted in pink. (**B1**,**B2**) The molecular surface representation of the TUBB4-GLUT1 complex after 50 ns. TUBB4 is shown in green, the cytoplasmic domain of GLUT1 is red and both the transmembrane and extracellular domains of GLUT1 are shown in grey. Residues at the top channel opening are shown in blue while residues at the opening near the transmembrane domain are colored in cyan. At this predicted position, TUBB4 (green) occludes the top opening in the GLUT1 channel. (**C**) Most probable poses of fasentin as predicted from the 1st (green), 2nd (yellow) and 3rd (pink) most populated clusters. (**D**) Interaction diagrams of the most probable poses of fasentin as predicted from the 1st (top) and 3rd (bottom) most populated clusters. (**E**) Docking model of CR-42-24 and TUBB.

The binding energy of TUBB4 to GLUT1 during the last 25 ns of simulations was calculated using MMGBSA in Ambertools14 and was predicted to be −27 kcal/moL. The calculated binding energy was decomposed to the individual residues contributing to binding (Table 1). The highest contributors to this interaction were residues Arg249-GLUT1 and Glu205-TUBB4, which had a binding energy of −18 kcal/moL. There were also strong interactions between Arg11-GLUT1 and Glu443-TUBB4, Asp236-GLUT1 and Lys379-TUBB4 as well as Glu462-GLUT1 and Arg391-TUBB4 with binding energies of −15, −12 and −9 kcal/moL, respectively (Table 1). We used clustering in Ambertools via the average-linkage algorithm to obtain the representative structure of the last 25 ns of simulation. Examination of this representative structure from MD simulations revealed that the cytoplasmic domain of TUBB4 had two openings: One at the top of the channel formed by residues Gln250, Phe395, Ser396, Gln397, Phe460, Glu462, Pro485 and Leu486 (Figure 4B.1) and another opening near the transmembrane domain of GLUT1 formed by residues Asn88, Arg212, Ile216, Glu247, Met251, Lys256, Val257 and Thr258 (Figure 4B.2). It is likely that the binding of TUBB4 to GLUT1 alters the transport of glucose through the channel at this docked position.

### 3.5. Binding Prediction Model of Fasentin and GLUT1

Results of the clustering analysis were used to identify the most probable binding poses. First, clusters with a large population should account for binding regions characterized by higher entropy, thus increasing the chance of finding the correct binding mode. At the same time, recent case studies performed on a set of various protein–ligand complexes revealed that if multiple programs such as Autodock, Vina and DOCK6 predict the same top binding pose within a 2.0 Å RMSD range, such a pose is likely to correspond to the correct binding mode [38,39]. Agreement of the three mentioned program on the same top pose (within a 2.0 Å RMSD range) leads to a success rate of 82% against 50–60% for the docking software alone [39]. Overall, clusters with a large population and involving multiple programs are more likely to account for the correct mode of binding.

Clustering analysis of the docked poses of fasentin (79 poses overall, 20 poses for Autodock, Vina and DOCK6, 19 poses for Glide SP) has revealed three clusters with large population (7 poses, 6 poses and 4 poses respectively). The most populated cluster (7 poses) includes poses predicted by all four programs used (Autodock, Vina, DOCK6 and GLIDE SP). The two binding modes associated with the second and third most populated clusters are also depicted. All three clusters account for poses located in the same area (Figure 4C). The first and second most populated clusters correspond to very similar poses characterized by the same acidic bond between the nitrogen group and residue Glu380 (Figure 4D). Figure 4E shows the docking results for the CR-42-04 with TUBB4.

### 3.6. TUBB4 Inhibition Silences GLUT1 Expression

**Figure 5:**
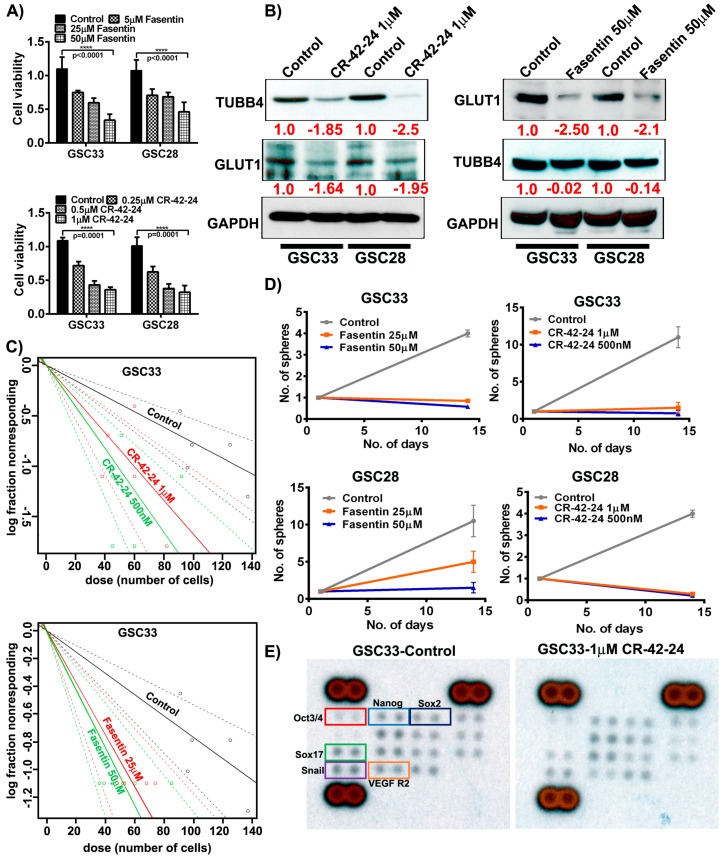
Targeting GLUT1 and TUBB4 reduces GSC cell growth and proliferation. (**A**) MTT analysis of increasing concentration of fasentin and CR-42-24 on GSC33 and GSC28. (**B**) Immunoblot analysis of GLUT1 and TUBB4 in GSC33 and GSC28 cells treated with 1µM CR-42-24 and 50 µM fasentin. GAPDH was used as a loading control. (**C**) Limiting dilution assays were performed on GSC33 cells treated with fasentin and CR-42-24 with increasing concentrations and increased number of cells. (**D**) Tumorsphere formation assay in GSC33 and GSC28 cells. (**E**) GSC33 cells treated with 1 µM CR-42-24 showed reduced levels of various stem cell factors when compared to the untreated cells. A human pluripotent stem cell antibody array (ARY010) was used in this study.

CR-42-24 is a novel colchicine-derived inhibitor developed by Dr. Jack Tsuzynski’s laboratory (University of Alberta, Canada). This inhibitor is a microtubule-inhibitor that demonstrates binding specific to TUBB4. Based on our modeling studies, PLA results and immunoprecipitation results, we believe that the blockade of TUBB4 may silence GLUT1 expression in GSC. To determine the optimal concentrations of both fasentin and CR-42-24, we performed MTT assays on both GSC33 and GSC28 cells. MTT assay at 50 µM fasentin treatment showed an IC50 value when compared to 5 µM and 25 µM fasentin treatments. CR-42-24 was effective at 1 µM compared to 0.25 µM and 0.5 µM treatments. For both these treatments, cells were incubated for 24 h (Figure 5A). Immunoblot analysis of GSC33 and GSC28 upon treatment with 1 µM CR-42-24, silenced the protein expression of GLUT1 and TUBB4 compared to untreated controls. Fasentin’s mechanism is well understood in the inhibition of GLUT1, however, its role in suppressing tumor growth has been vaguely discussed [40], but not yet within the context of diffuse gliomas, and certainly not in GBM. Immunoblot results indicate that GSC33 and GSC28 cells treated with 50 µM fasentin showed a loss of GLUT1 expression. Interestingly, no change was observed in the levels of TUBB4 expression (Figure 5B). Next, we asked if using these inhibitors contributed to the control of cell proliferation by using a limiting dilution assay. Fasentin (25 µM, 50 µM) and CR-42-24 (0.5 µM, 1 µM) treated GSC33 cells and its respective controls were plated in increasing number of cells as mentioned in methods section. The data obtained after 14 days showed that fasentin at 50 µM and CR-42-24 at 0.5 µM were effective in controlling the GSC growth (Figure 5C). Importantly, this limitation in growth was shown to affect cell growth proportionally to the concentration at which these GSC33 cells were treated. Tumorsphere formation assays performed on GSC33 and GSC28 cells upon treatment with fasentin at 50 µM and CR-42-24 at 1 µM showed similar effects from the limiting dilution assay as shown in Figure 5C. Interestingly, the untreated GSC showed significant tumorsphere formation by the 14th day (Figure 5D). We next verified if CR-42-24 at 1 µM could modulate the expression of stem cell markers in the GSC33. For this experiment we used the human pluripotent stem cell antibody array. The levels of Oct3/4, Nanog, Sox2, Sox17, Snail and VEGFR2 were significantly reduced in the CR-42-24 treatment when compared to the untreated controls (Figure 5E). Overall, our data confirmed that silencing TUBB4 or GLUT1 reduce GSC tumorsphere formation, self-renewal and proliferation in vitro.

## 4. Discussion

Our present work sets out to demonstrate the feasibility of GLUT1 as a druggable metabolic target in GBM. As previously mentioned, the perceived value of targeting the GLUT1 protein is that it is the first rate-limiting step in glycolysis and is disproportionately upregulated in higher grade tumor tissue. To confirm, we first show the definitive upregulation of GLUT1 in glioblastoma samples derived from patients from our own cohort and in comparison to that of the TCGA cohort dataset. We next demonstrated the feasibility of targeting this protein via in silico modeling. Finally, we showed that targeting GLUT1 as well as its associated protein, TUBB4, decreased cell viability, sphere formation ability and cell aggressiveness in vitro. We believe that these studies, when taken together, warrant further investigation into GLUT1 as a druggable target in GBM that should be examined in non-human in vivo models.

The Warburg effect, despite having been conceptualized by Otto von Warburg over 100 years ago, has received renewed interest of late in the search for valid drug targets in solid tumors including diffuse gliomas [41,42,43]. This strategy of chemotherapeutic drug development allows for the avoidance of extremely morbid side effects seen with most other chemotherapies. Temozolomide, the mainstay of treatment in GBM, causes significant myelosuppression and hepatotoxicity along with generalized weakness, fatigue and nausea [44]. It additionally allows for another adjuvant tool in the chemotherapeutic arsenal with regard to addressing the problem of chemoresistance, which develops in approximately 90% of recurrent GBMs [45]. Therefore, it makes intuitive sense that targeting of the transporters and enzymes that facilitate rate-limiting steps of glycolysis will yield positive outcomes.

Nishioka et al., were the first to publish on the prevalence of various glucose transport proteins in GBM, examining the mRNA expression of 18 human-derived samples [10]. They were the first to report on the prevalence of GLUT1, or the “erythrocyte-type” glucose transporter, as the most overexpressed transporter type in GBM, initially prompting our investigation into this protein. With a paucity of investigation throughout the 1990s and early 2000s, there has been a renewed interest in this protein for its prognostic significance in GBM patients. Recently, Bache et al., demonstrated an upregulation of GLUT1 mRNA expression in glioblastoma, proposing this along with other proteins, including hypoxia-inducible factor-1a (HIF-1a), VEGF and OCT4, as a marker of hypoxia-induced changes that define the metabolism of GBM [46]. Later Komaki and colleagues demonstrates GLUT1 expression in both pseudopalisaded and perivascular tumor cells, the two defining histologic cellular features of GBM patients [47]. As we suggest, these authors propose the enhancement of glycolysis as a potential mechanism for the correlation of GLUT1 expression with poorer prognosis in their selected patient population.

With regard to the tubulin family of proteins, there is additional literature available with respect to its involvement in glioblastoma. The earliest identified work on beta-tubulins in glioblastoma was published by Katsetos et al. in 2007 [48]. They demonstrate that beta and gamma tubulins are highly expressed in GBM, and to a lesser extent in lower-grade diffuse gliomas of astrocytic lineage. Bordji et al. in 2014 elucidates TUBB4 as a protein whose expression is propagated largely by the hypoxic cascade induced by the HIF family of proteins, (in this particular case, HIF-2a) [49]. Later, Phoa et al. published on the utility of novel small-molecule tubulin inhibitors as a means to drug glioblastoma cells [50]. Their compound, CMPD1, acts to inhibit tubulin polymerization and induce apoptosis in GBM cells in vitro, acting in a way similar to colchicine. However, the promising factor in this study is that small molecule inhibitors can bypass the blood–brain barrier far more readily than the large colchicine compound can. Importantly, this group demonstrated that CMPD1 works in EGFR-enhanced GBM cells, which tend to be more aggressive and radio/chemoresistant, supporting tubulins including TUBB4 as an important target for adjuvant therapy when chemoresistance to TMZ develops over time.

Much of our work in this project focused on modeling interactions between GLUT1 and fasentin as well as that between GLUT1 and TUBB4 using a consensus approach via the Autodock, Vina, DOCK6 and GLIDE SP software. Interestingly, we improved upon previously existing models of the interaction between GLUT1 and fasentin. The first paper published on the mechanism of fasentin by Wood and colleagues in 2008 utilized an algorithm FlexX, to demonstrate the in silico modeling of fasentin-GLUT1 interactions [12]. Their modeling utilized a GLUT1 model based on the crystalline structure of *Escherichia. coli* glycerol-3-phosphate antiporter. However, we were able to utilize the crystal structure of the human GLUT1 (PDB ID 4PYP), derived from Deng et al. [21].

## 5. Conclusions

In summary, we demonstrated that the glucose transporter GLUT1 was overexpressed in patient-derived human GBM tissue and its overexpression correlated with poor prognosis upon examination utilizing data mining of The Cancer Genome Atlas. We then worked to establish the association between GLUT1 and TUBB4, a protein that we believed might be involved in membrane trafficking. We demonstrated the likelihood of this association in both human-derived GBM samples via mass spectrometry as well as in silico modeling, which demonstrated favorable binding energies between the two molecules. Then, we elucidated the mechanism of fasentin, a GLUT1-specific antagonist, via in silico modeling, while showing that inhibition of TUBB4 with a novel colchicine derivative specific to this isozyme also knocks down GLUT1 expression in both GSC33 and GSC28 cell lines.

## Figures and Tables

**Table 1:** Binding energies calculated for GLUT1 and TUBB4. The values are expressed in kcal/moL.

GLUT1	TUBB4	Binding Energy (kcal/moL)
Lys 7	Glu443	−8
Arg 11	Glu443	−15
Asn 219	Arg213	−3
Arg 223	Ala445	−8
Lys 229	Glu441	−4
Lys 230	Glu441	−4
Lys 230	Glu442	−4
Lys 230	Ala445	−4
Asp 236	Lys379	−12
Lys 245	Glu205	−7
Glu 246	Lys174	−3
Arg 249	Glu205	−18
Asp 461	Arg390	−7
Asp 461	Arg391	−8
Glu 462	Arg391	−9

## Supplementary Materials

The following are available online at https://www.mdpi.com/2072-6694/11/9/1308/s1.
