# Supplementary material for: GLUT1 and TUBB4 in Glioblastoma Could be Efficacious Targets
Source: Cancers (Basel). 2019 Sep 5;11(9):1308. doi: 10.3390/cancers11091308 (PMC6771132; doi:10.3390/cancers11091308)
Supplement: Supplementary file 1 [file cancers-11-01308-s001.pdf]

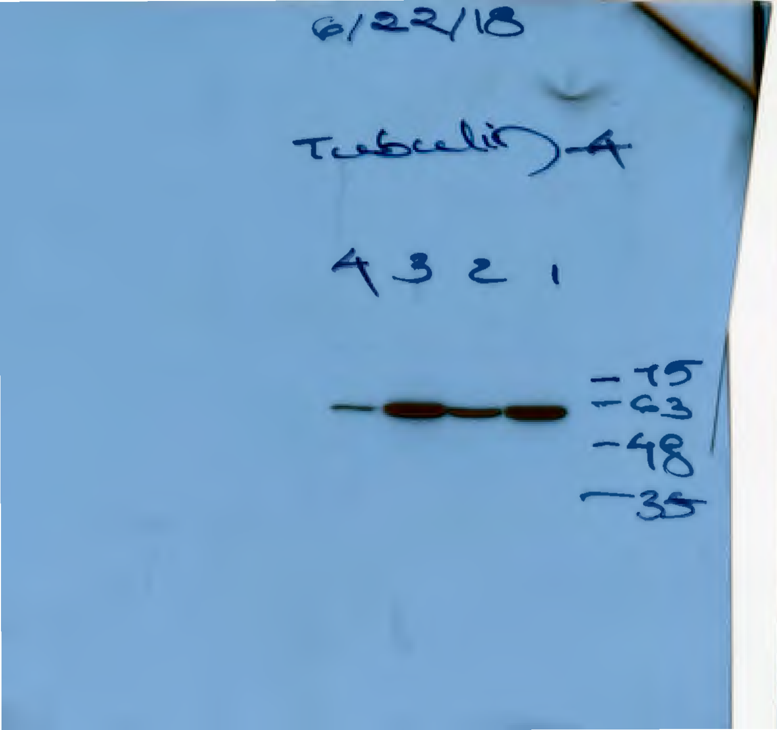
6/22/18
Tubulin-A
4 3 2 1
75
63
48
35

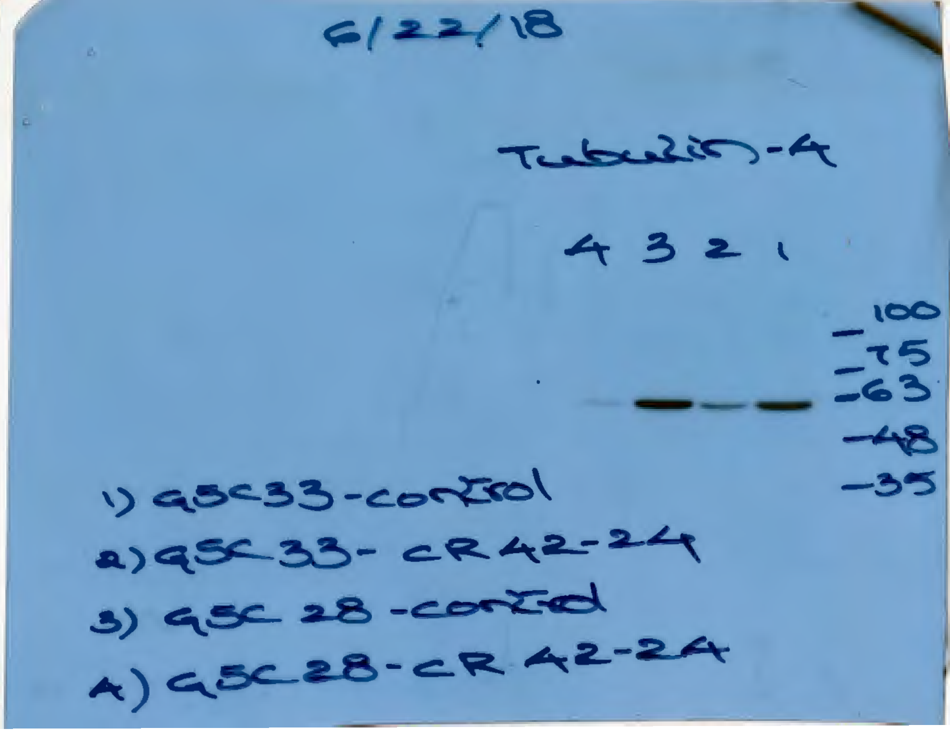
6/22/18
Tubulin-A
4 3 2 1
100
75
63
48
35
1) Q5C33-control
2) Q5C33- CR42-24
3) Q5C 28 -control
4) Q5C28-CR 42-24

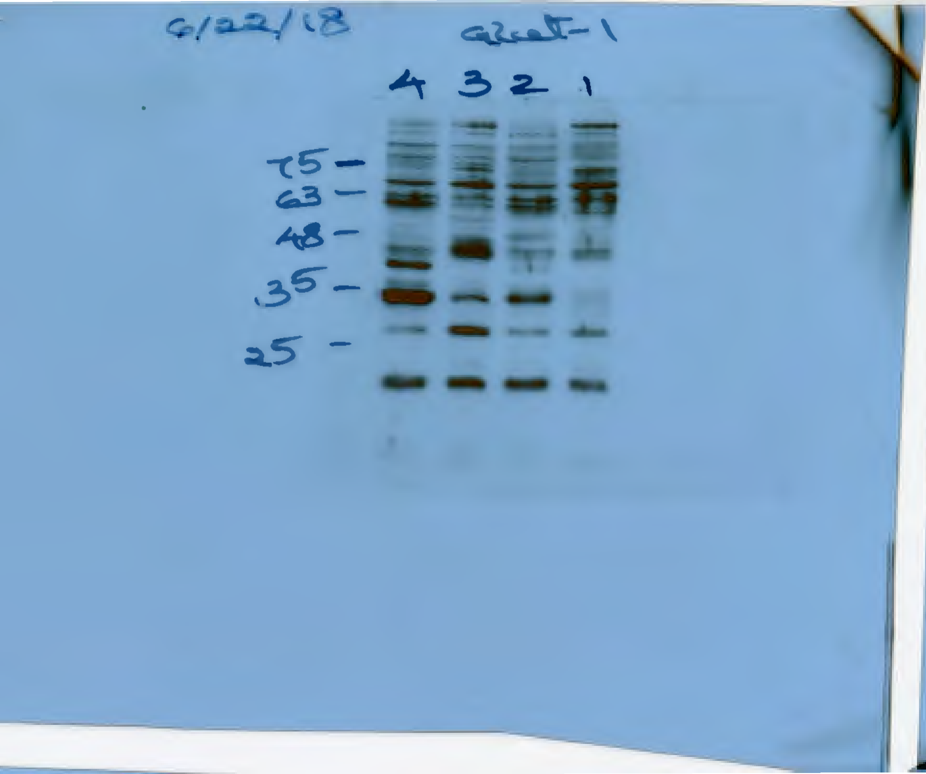

6/22/18
4 3 2 1
75 -
63 -
48 -
35 -
25 -

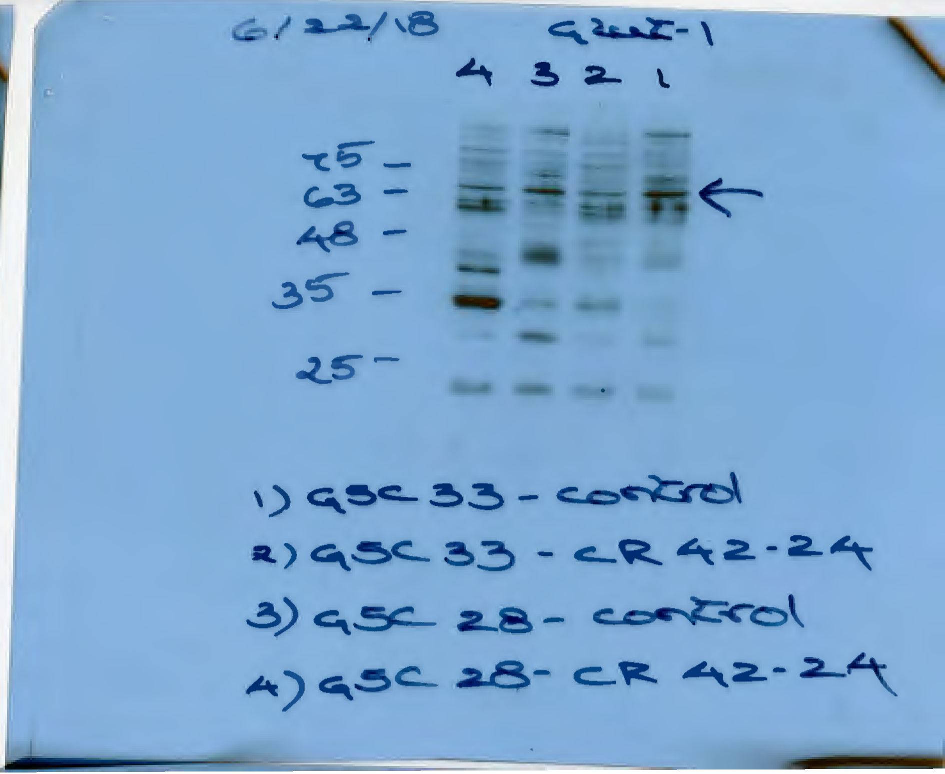

6/22/18
4 3 2 1
75 -
63 -
48 -
35 -
25 -
1) GSC 33 - control
2) GSC 33 - CR 42-24
3) GSC 28 - control
4) GSC 28 - CR 42-24

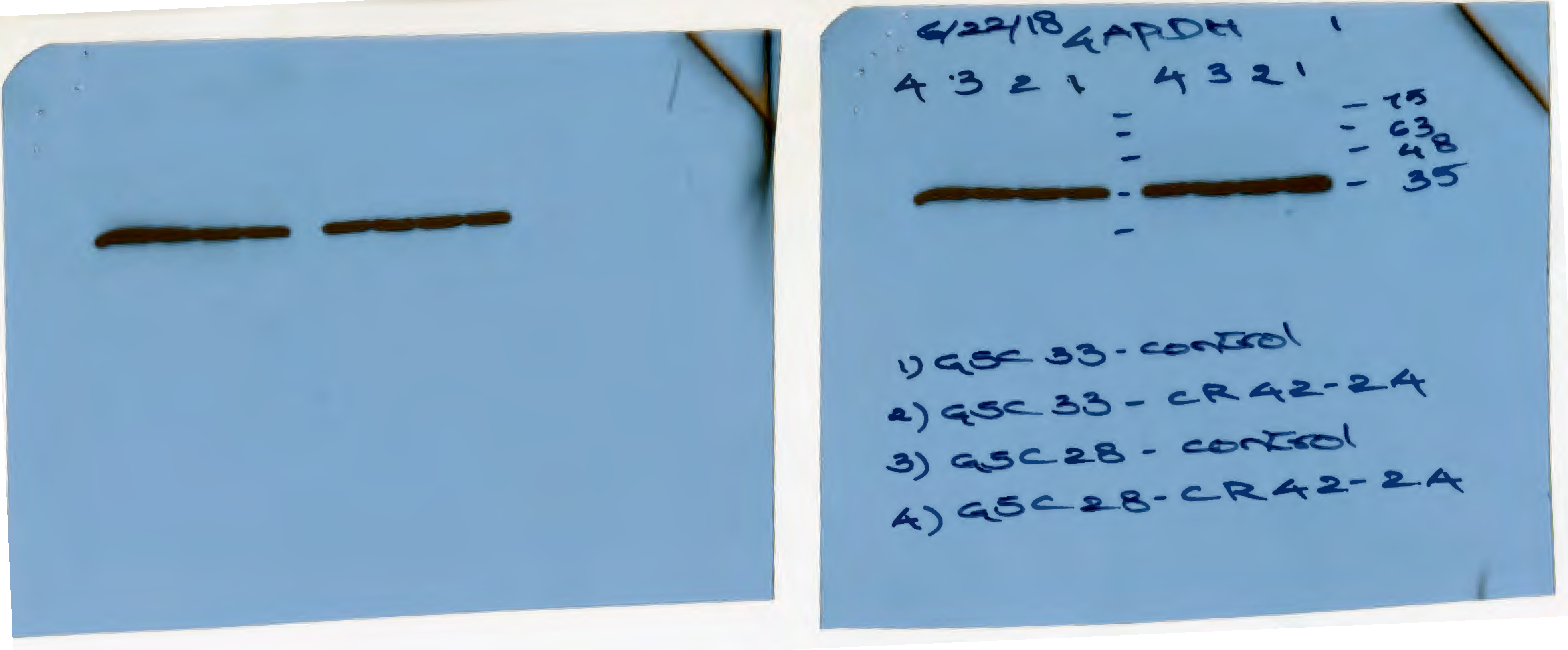
6/22/18 GAPDH
4 3 2 1 4 3 2 1
75
63
48
35
1) GSC 33 - control
2) GSC 33 - CR 42-2A
3) GSC 28 - control
4) GSC 28 - CR 42-2A

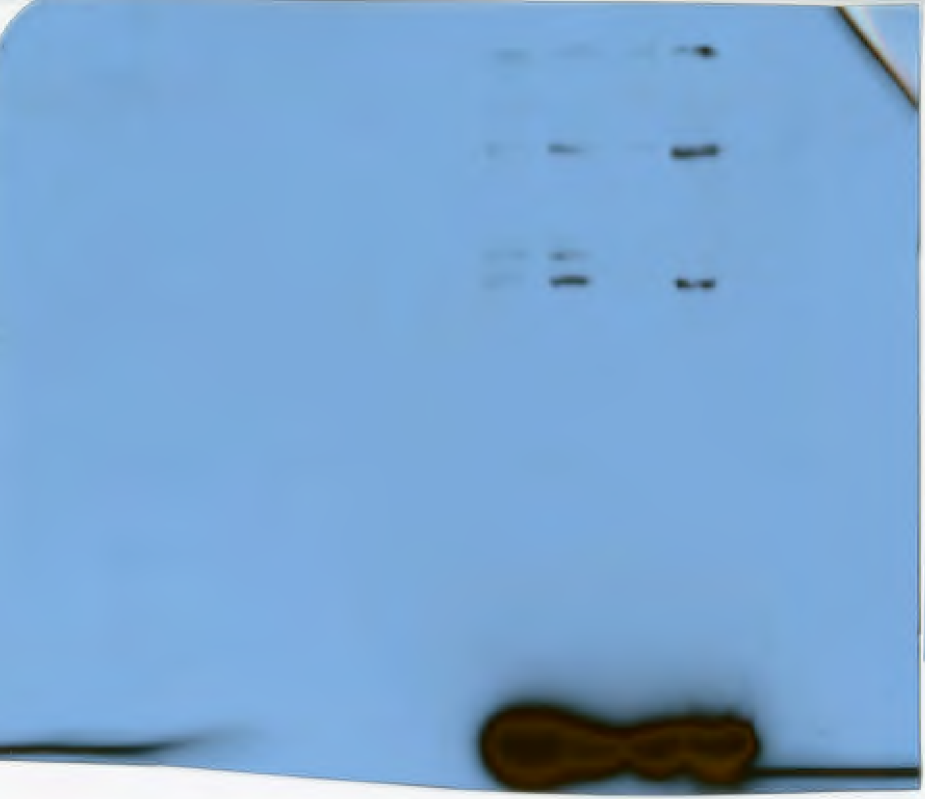

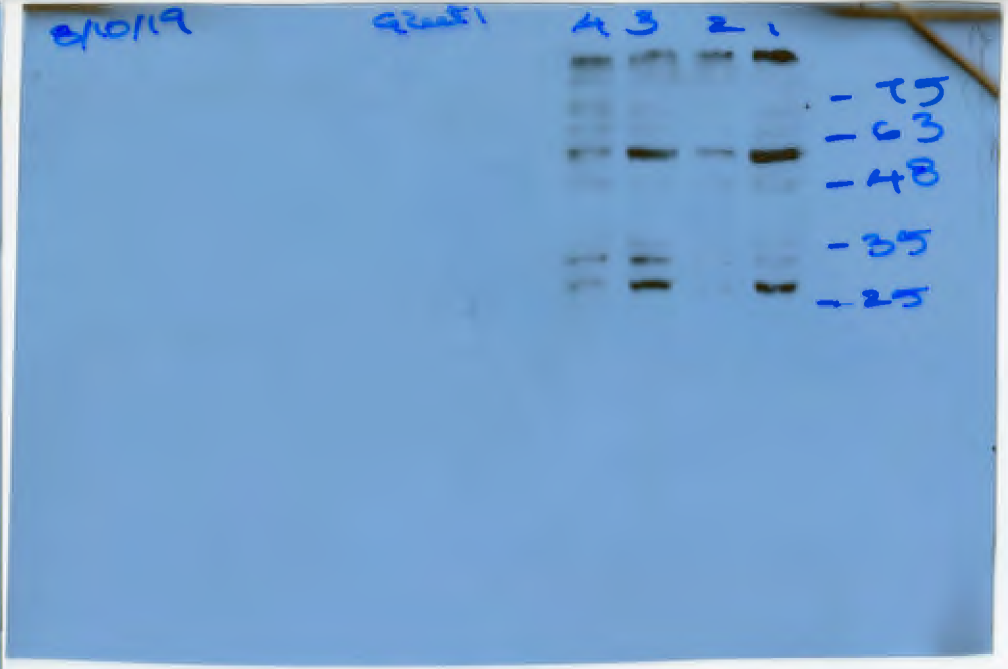
8/10/19
4 3 2 1
- 75
- 63
- 48
- 35
- 25

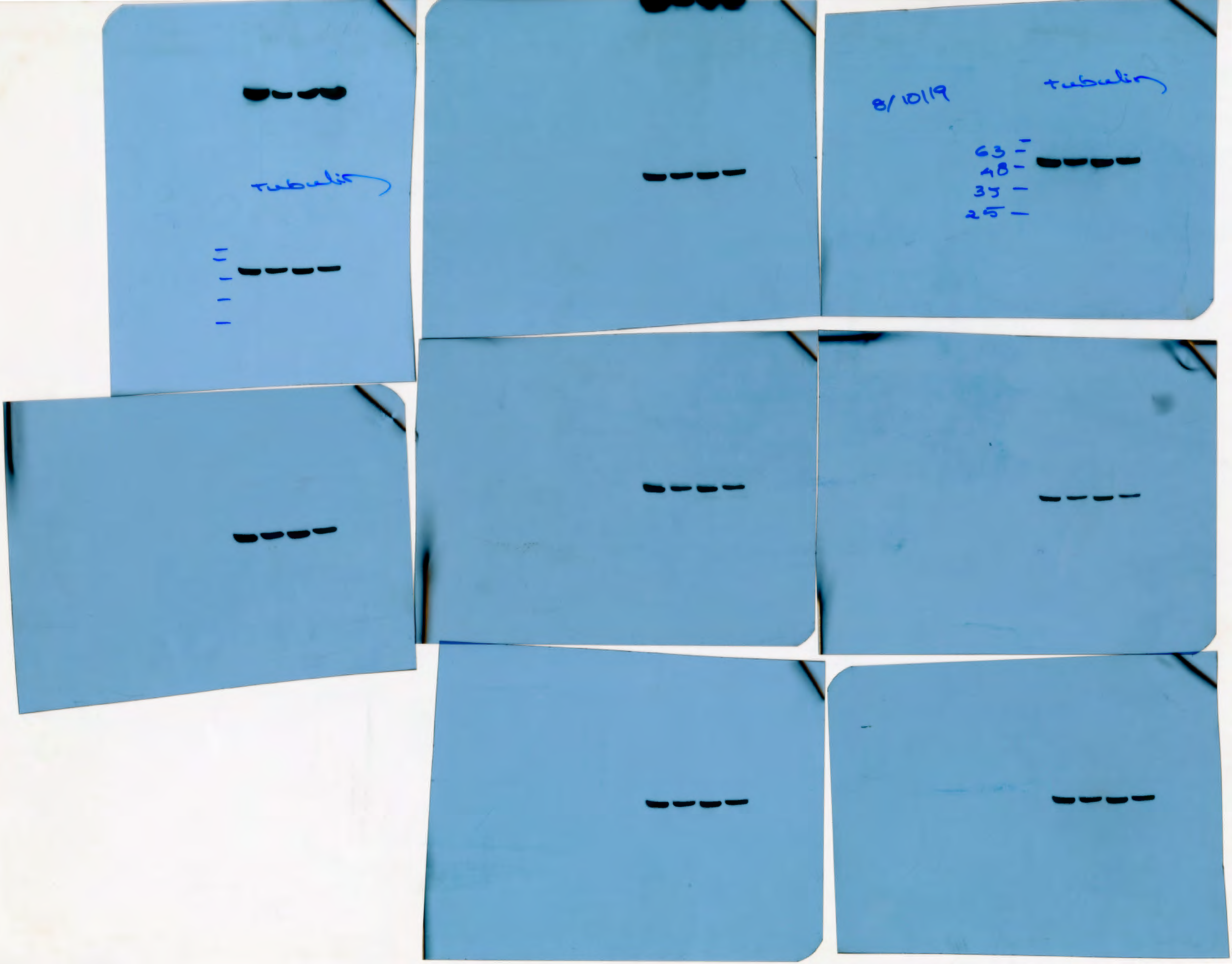

tubulin
8/10/19
tubulin
63
48
35
25

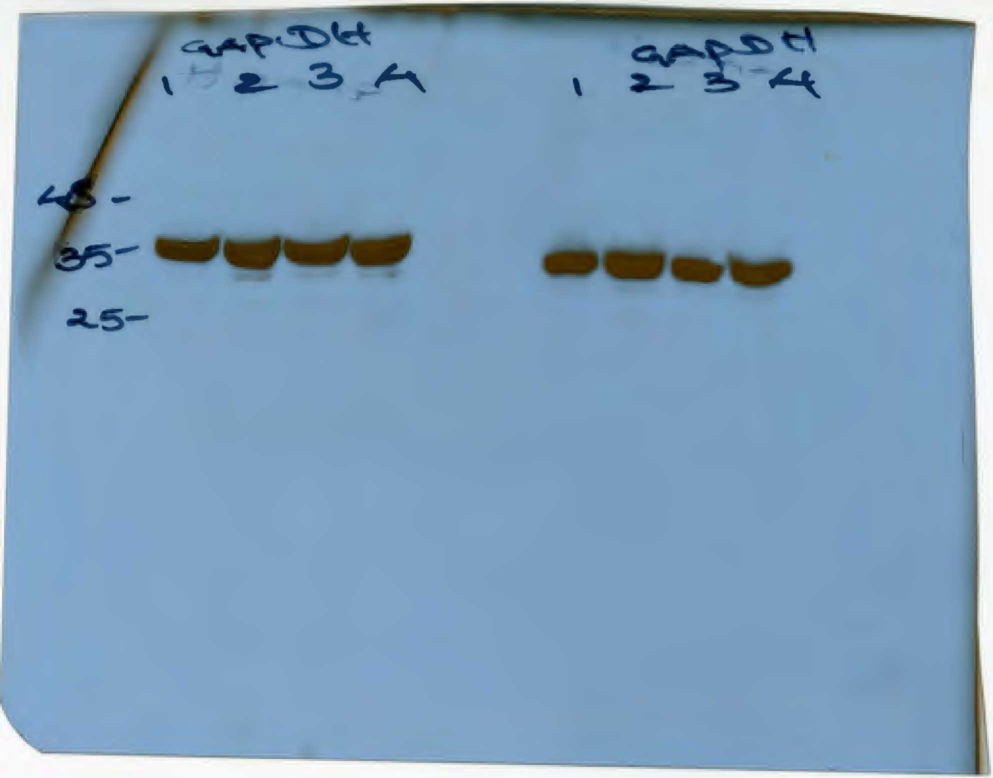
GAPDH
1 2 3 4
GAPDH
1 2 3 4
35-
25-

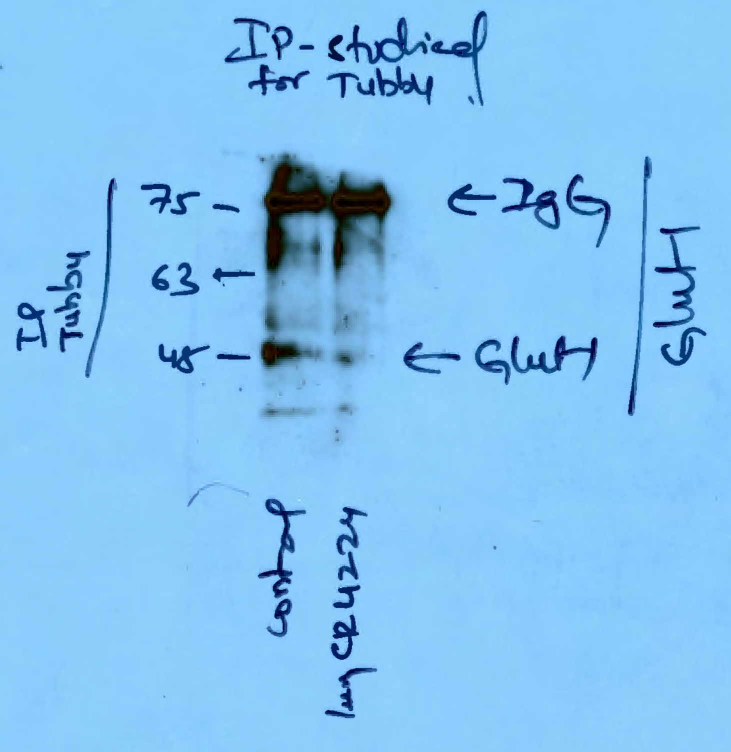
IP- studied for Tubby
75 –
63 ←
48 –
← IgG
control

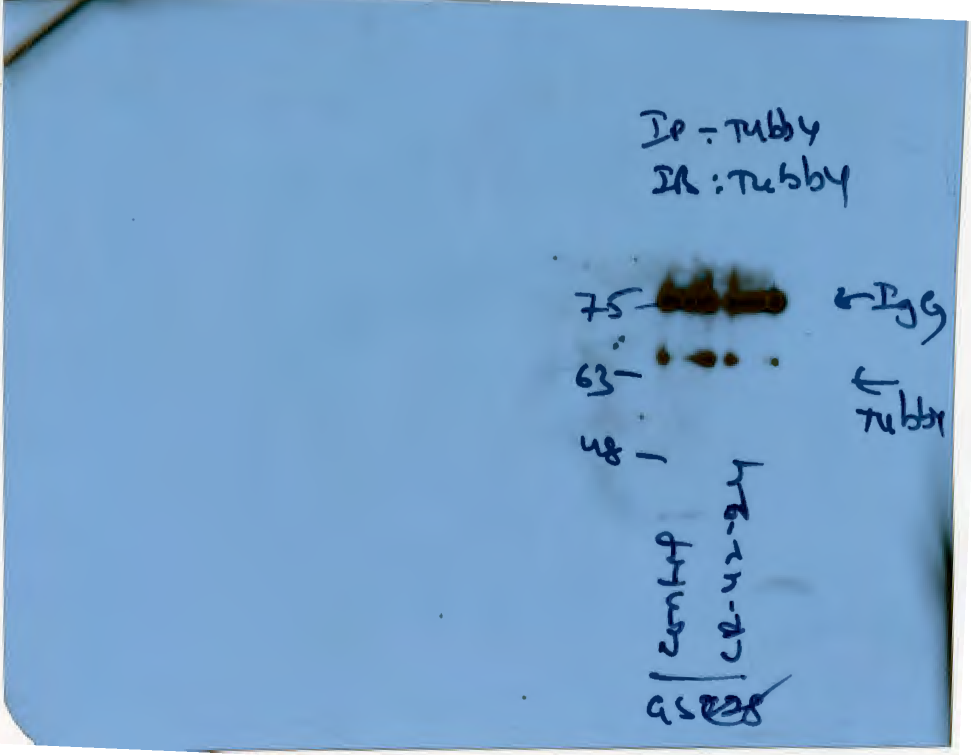
IP : Tubby
IB : Tubby
75
63
48
IgG
Tubby

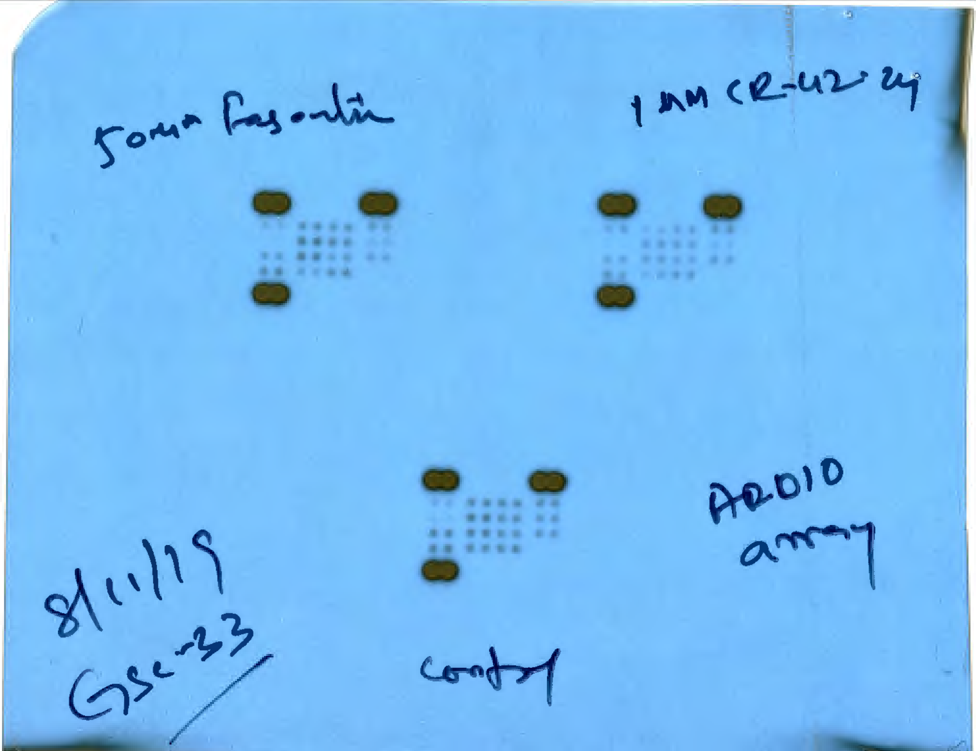
1 nM CR-42-24
8/11/19
GSC-33
control
